# Draft Genome Sequence of *Lactobacillus plantarum* CRL1506, an Immunomodulatory Strain Isolated from Goat Milk

**DOI:** 10.1128/genomeA.00108-16

**Published:** 2016-03-10

**Authors:** Lucila Saavedra, Elvira María Hebert, Leonardo Albarracin, Susana Salva, Susana Alvarez, Haruki Kitazawa, Julio Villena

**Affiliations:** a Reference Centre for Lactobacilli (CERELA-CONICET), San Miguel de Tucumán, Tucumán, Argentina; b Food and Feed Immunology Group, Laboratory of Animal Products Chemistry, Graduate School of Agricultural Science, Tohoku University, Sendai, Japan; c Livestock Immunology Unit, International Education and Research Center for Food Agricultural Immunology (CFAI), Graduate School of Agricultural Science, Tohoku University, Sendai, Japan

## Abstract

This report describes a draft genome sequence of *Lactobacillus plantarum* CRL1506, a probiotic strain with immunomodulatory properties isolated from goat milk. The reads generated by a whole-genome shotgun (WGS) strategy on an Illumina MiSeq sequencer were assembled into contigs with a total size of 3,228,096 bp. The draft genome sequence of *L. plantarum* CRL1506 will be useful for further studies of specific genetic features of this strain and for understanding the mechanisms of its immunobiotic properties.

## GENOME ANNOUNCEMENT

*Lactobacillus plantarum* CRL1506 (formerly *Lactobacillus rhamnosus*) was isolated from goat milk (1). *In vitro* and *in vivo* studies demonstrated that this strain increases resistance against bacterial and viral pathogens (2–4) and that those effects are related to its capacity to improve mucosal immune responses. *L. plantarum* CRL1506 differentially modulates the production of pro- and anti-inflammatory cytokines and type I interferons in mucosal epithelial cells and antigen-presenting cells, including macrophages and dendritic cells (5, 6). Those studies suggest that this strain is an excellent candidate as a probiotic to prevent mucosal infections.

Here we present a draft genome sequence of *L. plantarum* CRL1506 that was sequenced using a whole-genome shotgun (WGS) strategy on an Illumina MiSeq sequencer. In total, 5,675,109 paired-end sequenced reads with lengths of 150 bp were obtained, which yielded 1.04 Gb of total sequenced bases with 527-fold coverage. Quality filtered reads were assembled using the Ngen (DNASTAR) assembler, giving 14 contigs. The longest contig was 1,048,781 bp and the shortest 22.273 bp. The functional annotation of predicted genes was achieved using the RAST server and the NCBI Prokaryotic Genome Annotation Pipeline (PGAP) (1). tRNAs and rRNAs were identified by tRNAscan-SE and RNAmmer, respectively (7, 8).

The draft genome of *L. plantarum* CRL 1506 consists of 3,228,096 bp with a mean G+C content of 44.55%. A total of 2,903 coding sequences (CDS), 67 structural tRNAs, and 13 rRNA were predicted. Among all CDS, 2,041 (70%) were assigned to known protein functions while 862 (30%) remain as hypothetical proteins. Additionally, there are 331 RAST subsystems represented in the genome, which represent only 43% of the assigned sequences.

The CRL1506 genome contains two genes encoding mucus-binding proteins (MBP*)* with 100% identity to *MBP* found in other *L. plantarum* strains (WP_027821315.1 and WP_058011789.1). In addition, genes encoding homologues of bacteriocin ABC-transporters and immunity proteins, as well as a structural gene with 100% identity to plantaricin-A bacteriocin (WP_003641979.1), were found in the CRL1506 genome, suggesting a potential of this strain to produce antimicrobial compounds. Similar to other strains of the species, *L. plantarum* CRL1506 contains genes responsible for exopolysaccharide biosynthesis.

The draft genome sequence of *L. plantarum* CRL1506 will be useful for further studies of specific genetic features of this strain and for understanding the mechanisms of its immunobiotic properties.

### Nucleotide sequence accession numbers.

This whole-genome shotgun project has been deposited at DDBJ/EMBL/GenBank under the accession number LNCP00000000. The version described in this paper is version LNCP00000000.1
